# A novel and practical cardiovascular magnetic resonance method to quantify mitral annular excursion and recoil applied to hypertrophic cardiomyopathy

**DOI:** 10.1186/1532-429X-16-35

**Published:** 2014-05-20

**Authors:** Shahryar G Saba, Sohae Chung, Sharath Bhagavatula, Robert Donnino, Monvadi B Srichai, Muhamed Saric, Stuart D Katz, Leon Axel

**Affiliations:** 1 Department of Medicine, Leon H. Charney Division of Cardiology, New York University Langone Medical Center, 550 First Avenue, New York, NY 10016, USA; 2 Department of Radiology, Center for Biomedical Imaging, New York University Langone Medical Center, 660 First Avenue, Room 411, New York, NY 10016, USA; 3 Current affiliation: National Heart, Lung, and Blood Institute, National Institutes of Health, Bethesda, MD 20892, USA; 4 Current affiliation: Medstar Heart Institute, Medstar Georgetown University Hospital, Washington DC 20007, USA

**Keywords:** Cardiovascular magnetic resonance, Mitral annular motion, Left ventricular function, Hypertrophic cardiomyopathy

## Abstract

**Background:**

We have developed a novel and practical cardiovascular magnetic resonance (CMR) technique to evaluate left ventricular (LV) mitral annular motion by tracking the atrioventricular junction (AVJ). To test AVJ motion analysis as a metric for LV function, we compared AVJ motion variables between patients with hypertrophic cardiomyopathy (HCM), a group with recognized systolic and diastolic dysfunction, and healthy volunteers.

**Methods:**

We retrospectively evaluated 24 HCM patients with normal ejection fractions (EF) and 14 healthy volunteers. Using the 4-chamber view cine images, we tracked the longitudinal motion of the lateral and septal AVJ at 25 time points during the cardiac cycle. Based on AVJ displacement versus time, we calculated maximum AVJ displacement (MD) and velocity in early diastole (MVED), velocity in diastasis (VDS) and the composite index VDS/MVED.

**Results:**

Patients with HCM showed significantly slower median lateral and septal AVJ recoil velocities during early diastole, but faster velocities in diastasis. We observed a 16-fold difference in VDS/MVED at the lateral AVJ [median 0.141, interquartile range (IQR) 0.073, 0.166 versus 0.009 IQR -0.006, 0.037, P < 0.001]. Patients with HCM also demonstrated significantly less mitral annular excursion at both the septal and lateral AVJ. Performed offline, AVJ motion analysis took approximately 10 minutes per subject.

**Conclusions:**

Atrioventricular junction motion analysis provides a practical and novel CMR method to assess mitral annular motion. In this proof of concept study we found highly statistically significant differences in mitral annular excursion and recoil between HCM patients and healthy volunteers.

## Background

Atrioventricular plane displacement, a measure of longitudinal left ventricular (LV) function, accounts for approximately 60% of the stroke volume [1]. Reduced mitral annular plane systolic excursion (MAPSE), measured by M-mode echocardiography, provides a sensitive, early marker of systolic dysfunction in hypertensive patients with preserved EF, and can diagnose heart failure with preserved EF [2]. Mitral annular velocity (E’), measured in early diastole by tissue Doppler echocardiography, incrementally predicts cardiac mortality beyond clinical data and standard echocardiographic measures [3,4]. At the present time, however, there is still no simple, validated cardiovascular magnetic resonance (CMR) technique to measure mitral annular excursion and recoil.

In the current study we investigated the movement of the atrioventricular junction (AVJ) throughout the cardiac cycle. We define the septal and lateral AVJ as myocardial points at the corresponding left atrial-ventricular junctions in long axis cine images. The maximum longitudinal displacement of the AVJ in systole corresponds to peak mitral annular excursion. The maximum velocity of the AVJ in early diastole reflects peak mitral annulus recoil velocity during passive LV filling. We previously showed that AVJ MVED represents a statistically significant CMR correlate of tissue Doppler echocardiography E’ [5], and we have confirmed these findings in a separate cohort of patients from a different institution. We analyzed 27 patients who underwent both studies within 24 hours, and again found a statistically significant (p = 0.001) correlation between CMR-derived MVED and E’ (r = 0.624) [6].

To test our hypothesis that hearts with systolic and diastolic dysfunction, despite preserved EF, would demonstrate abnormal AVJ motion, we performed our analysis on patients with HCM. With the highest prevalence of any genetic cardiac disorder, HCM affects approximately 1 in 500 individuals and may cause considerable morbidity and mortality [7]. The disease is known to be associated with diastolic [8] and regional LV systolic dysfunction [9].

Atrioventricular junction motion tracking software, developed in our laboratory, measures the longitudinal position of the AVJ on routinely obtained long axis cine images as a function of time during the cardiac cycle. Analysis of steady state free precession (SSFP) cine images provides better temporal resolution compared to conventional phase-contrast imaging techniques [10] used to evaluate myocardial velocity, without adding to acquisition time. Performed offline, in an efficient manner, AVJ motion analysis quantifies mitral annular excursion and recoil.

## Methods

### Study population

We retrospectively identified 30 consecutive patients between the ages of 21 and 64 with known or suspected HCM who underwent CMR as part of their routine clinical assessment at one of three affiliated hospitals (New York University Langone Medical Center, Bellevue Hospital Center, and the Manhattan Veteran’s Affairs Hospital) between February 2006 and July 2011. Eligible patients demonstrated LV hypertrophy (LVH) of at least 15 mm, not attributable to another etiology such as hypertension or diabetes, in a minimum of one myocardial segment. We excluded 5 patients without significant LVH, and one patient with concomitant evidence of left ventricular non-compaction. Prior to inception of the current study, healthy volunteers with no significant past medical history had been recruited to establish baseline AVJ motion variable values. A total of 14 healthy volunteers without hypertension, diabetes mellitus, coronary artery disease or other significant past medical history, and all with normal CMR examinations, were matched for mean age and used for comparison with the 24 HCM patients. Institutional review board approval was granted, and informed consent was obtained from all healthy volunteers.

### CMR protocol

As part of routine clinical protocol, HCM patients underwent CMR evaluation on 1.5 T or 3 T systems (Avanto, Tim Trio, Verio, Siemens, Erlangen, Germany) using a torso phased-array receiver coil. Two-dimensional SSFP pulse sequence cine imaging with electrocardiographic gating was performed in multiplanar short and long axis views, for evaluation of LV wall thickness, mass, segmental wall motion and global systolic function. Contrast-enhanced T1-weighted inversion-recovery late gadolinium enhancement (LGE) imaging was acquired for evaluation of myocardial fibrosis. Delayed imaging was performed 10 minutes after patients received intravenous gadolinium-diethylenetriamine penta-acetic acid (0.15 mmol/kg) contrast agent. Quantitative measurements of LV volumes, EF and mass, as well as qualitative assessments of LA size, severity of mitral regurgitation and systolic anterior motion of the mitral valve leaflets were performed. Post-processing, including the measurement of LV wall thickness, was performed on each segment of a 17-segment LV model, excluding the apical cap.

Healthy volunteers were imaged with a 3 T CMR system (Tim Trio, Siemens, Erlangen, Germany), using standard phased-array coils. Typical imaging parameters for patients and healthy volunteers included: TR = 2.4 ms, TE = 1.4 ms, flip angle 51°, slice thickness = 6 mm, in-plane spatial resolution = 1.6 mm × 1.6 mm, receiver bandwidth = 930 Hz/pixel, 20–30 phases (average 25 phases) per cardiac cycle and temporal resolution ~ 45 ms. To cover late diastolic filling, image acquisition was performed throughout the cardiac cycle with retrospectively gated reconstruction.

### Image and data analyses

**Figure 1:**
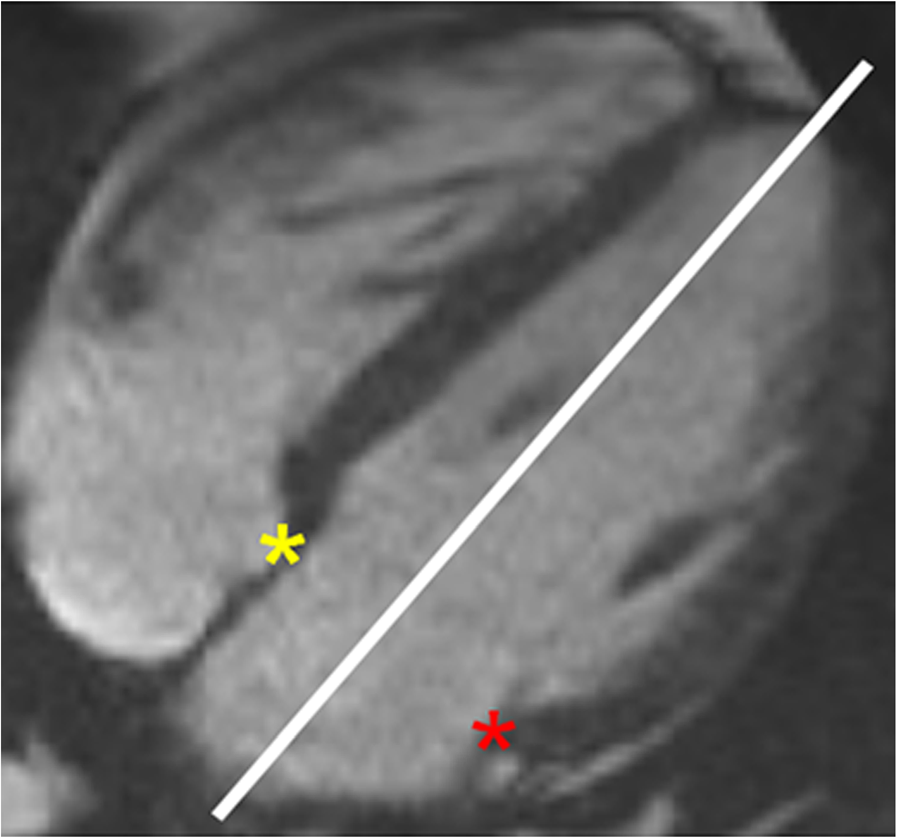
**Septal and lateral AVJ.** Yellow and red asterisks mark the septal and lateral AVJ, respectively, on this 4-chamber view. A reference line for longitudinal AVJ motion bisects the LV between the apex and the midpoint of the mitral annulus. AVJ = atrioventricular junction; LV = left ventricle.

Atrioventricular motion was measured at the septal and lateral junction points between the left atrium and ventricle in the 4-chamber cine view. A reference line was placed through the LV apex and the midpoint of the mitral annulus (Figure 1). The AVJ demonstrated an arc-like motion with longitudinal (toward the apex) and radial (toward the center of the mitral annulus) components. The longitudinal displacement was effectively measured as the distance between a perpendicular line through the reference line and AVJ at end diastole, and a perpendicular line through the reference line and AVJ at the given time point in the cardiac cycle. This distance was interactively measured over the cardiac cycle, using our custom-written (MATLAB, Natick, MA) AVJ motion-tracking software. Standardized atrioventricular junction motion analysis was performed off-line and took approximately 10 minutes per subject.

**Figure 2:**
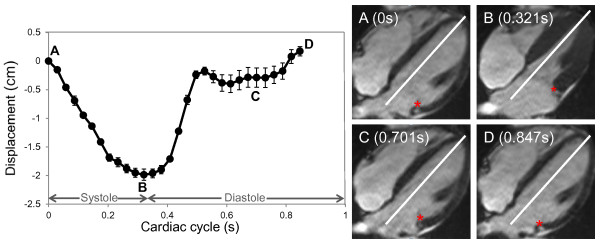
**AVJ displacement-versus-time plot and images.** Atrioventricular junction motion tracking software interactively measures the longitudinal AVJ position at multiple time points during the cardiac cycle. Error bars represent one standard deviation above and below the mean of multiple independent measurements made on a representative healthy volunteer. Lateral AVJ position, marked with a red asterisk, shown at the start of systole **(A)**, end systole **(B)**, diastasis **(C)** and end diastole **(D)** for a representative 4-chamber cine image set. AVJ = atrioventricular junction.

**Figure 3:**
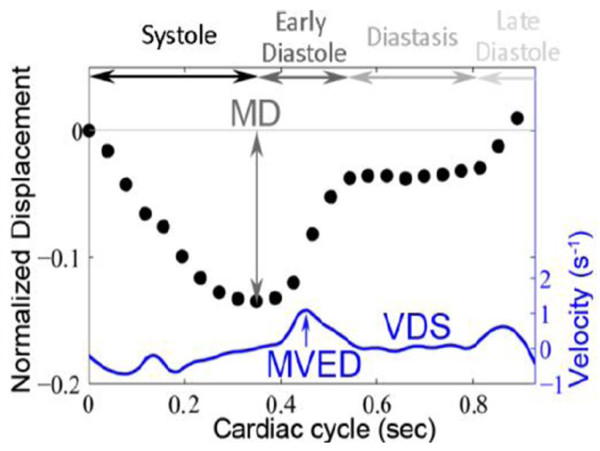
**AVJ normalized velocity-versus-time plot.** Derived from the AVJ normalized displacement-versus-time plot (dotted), the AVJ normalized velocity-versus-time plot (blue) shows the calculated AVJ motion variables MVED and VDS, as indicated. The motion tracking program directly calculates the MD from the AVJ normalized displacement-versus-time plot. AVJ = atrioventricular junction; MD = maximum displacement; MVED = maximum velocity early diastole; VDS = velocity diastasis.

All distances measured were divided by the longitudinal length of the LV at end diastole to provide an approximate correction for individual heart sizes. The AVJ was identified and marked on each of the cine phases obtained (Figure 2), and several motion variables were calculated (Figure 3). Based on the resulting time-versus-displacement plot, the AVJ motion variable MD (maximum displacement normalized by end-diastolic length) was determined. The slopes between sequential points of the normalized displacement-versus-time curve were calculated and plotted, resulting in a normalized velocity-versus-time curve. The maximum normalized velocity of the AVJ during early passive mitral filling (MVED), and the best-fit line of normalized AVJ velocity in diastasis (VDS) were also calculated. In order to establish intra-study reliability, AVJ motion analysis was also performed in a previously described cohort of patients referred for CMR [5] using both 3- and 4-chamber long axis cine images.

### Statistical methods

The continuous AVJ motion variables are presented as medians and IQR. Comparisons between AVJ motion variables in healthy volunteers and HCM patients were made using the Mann–Whitney *U* test. Box-and-whisker plots were generated to graphically represent differences between the two groups. Inter-reader variability analysis was performed on the healthy volunteer cohort data obtained by two independent observers. To assess intra-study reproducibility, intraclass correlation coefficients were also obtained in a separate cohort of patients that was previously described [5]. SPSS Statistics Version 20 was used to carry out all statistical analysis.

## Results

### Patient characteristics and CMR parameters

The mean age of the HCM patients and healthy volunteers in the study was 45 (range 25–59) and 46 (range 31–64), respectively. The mean thickness of the maximally hypertrophied segment was found to be 23 mm (range 16–32 mm). On average, each patient had 4.5 segments of myocardial hypertrophy (range 2–9). All HCM patients were found to have normal or increased EF (Table 1). Of the 24 patients analyzed, 18 (75%) patients demonstrated asymmetric septal hypertrophy, four (17%), mixed apical and asymmetric septal hypertrophy, and two (8%), concentric LVH. The most common location of maximum hypertrophy was found to be in the basal anteroseptum (9 patients, 38%). None of the segments in the basal or mid lateral wall or the entire inferior wall displayed maximum hypertrophy. Similarly, the most common location of any LVH > 15 mm was the basal anteroseptum (19 patients, 79%). Although there were areas of hypertrophy noted in the anterolateral, inferolateral and inferior segments, the majority were localized to the basal and mid anterior and septal segments.

**Table 1:** HCM clinical and CMR characteristics

	**Mean (Range or % Patients)**
**Age (years)**	45 (25–59)
**Women**	4 (17)
BMI^* ^**(kg/m**^**2**^**)**	26 (14–47)
**Maximum hypertrophy (mm)**	23 (16–32)
**# Hypertrophied segments ≥ 15 mm**	4.5 (2–9)
**Presence SAM**^**‡**^	13 (62)
**Presence MR**^**§**^	13 (57)
**Presence LGE**^**||**^	20 (83)
**LV EF**^**¶ **^**(%)**	66 (58–81)
**LVEDV**^**# **^**(mL)**	160 (56–285)
LVEDVI^** ^**(mL/m**^**2**^**)**	80 (43–134)
**Presence LA**^**† **^**dilation**	14 (70)

^*^BMI = body mass index; ^†^LA = left atrium; ^#^LVEDV = left ventricle end diastolic volume; ^**^LVEDI = left ventricle end diastolic volume index; ^¶^LV EF = left ventricle ejection fraction; ^||^LGE = late gadolinium enhancement; ^§^MR = mitral regurgitation; ^‡^SAM = systolic anterior motion.

### Late gadolinium enhancement

Of the 24 patients analyzed, 20 (83%) demonstrated regions of LGE consistent with myocardial fibrosis. None of the lateral wall segments showed evidence of LGE. The most common location of fibrosis was found to be in the basal anteroseptum (14 patients, 58%), followed by the mid anteroseptum (12 patients, 50%). The majority of hypertrophied segments demonstrated LGE. Of the 19 patients with basal anteroseptal hypertrophy, 14 (74%) were found to have LGE.

### AVJ motion analysis

**Figure 4:**
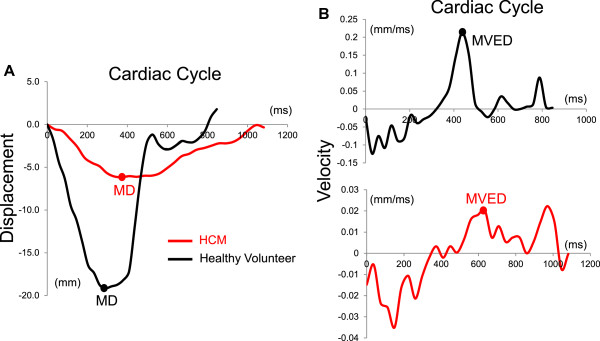
**HCM and healthy volunteer AVJ displacement-versus-time and velocity-versus-time plots. A**. Longitudinal AVJ displacement plotted as a function of time during the cardiac cycle in a representative HCM patient (red) and healthy volunteer (black). The healthy volunteer demonstrates a significantly greater MD compared to the patient with HCM. **B**. Derived from the displacement-versus-time curves, the velocity-versus-time plots show a significantly decreased recoil velocity during early diastole (MVED) in the HCM patient compared to the healthy volunteer. AVJ = atrioventricular junction; HCM = hypertrophic cardiomyopathy; MD = maximum displacement; MVED = maximum velocity early diastole.

**Figure 5:**
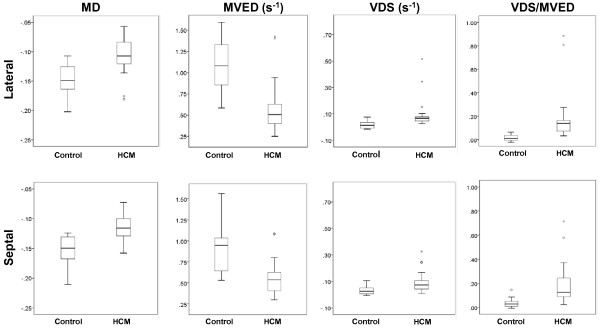
**Box plots of AVJ motion variables in HCM patients and healthy volunteers.** The box plots display the sample minimum (lower whisker), lower quartile (lower box subdivision), median (horizontal band), upper quartile (upper box subdivision), and sample maximum (upper whisker) for each of the AVJ motion variables MD, MVED, VDS and VDS/MVED at the lateral and septal AVJs in healthy volunteers and HCM patients. Circles indicate outliers. P ≤ 0.006 for all. AVJ = atrioventricular junction; HCM = hypertrophic cardiomyopathy; MD = maximum displacement; MVED = maximum velocity early diastole; VDS = velocity diastasis.

**Figure 6:**
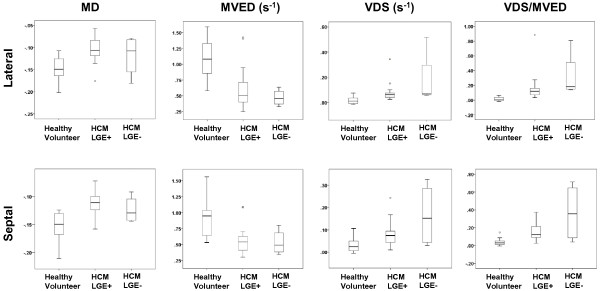
**Box plots of AVJ motion variables in HCM patients with and without LGE compared to healthy volunteers.** The box plots display the sample minimum (lower whisker), lower quartile (lower box subdivision), median (horizontal band), upper quartile (upper box subdivision), and sample maximum (upper whisker) for each of the AVJ motion variables MD, MVED, VDS and VDS/MVED at the lateral and septal AVJ in healthy volunteers and HCM patients with and without LGE. Circles indicate outliers. Both HCM patients with and without LGE demonstrated statistically significant differences (P < 0.05) in MVED, VDS and VDS/MVED at both the lateral and septal AVJ compared to healthy volunteers. AVJ = atrioventricular junction; HCM = hypertrophic cardiomyopathy; LGE = late gadolinium enhancement; MD = maximum displacement; MVED = maximum velocity early diastole; VDS = velocity diastasis.

We observed highly significant statistical differences in all three AVJ motion variables (MD, MVED, and VDS) and the composite index VDS/MVED, in patients with HCM when compared to healthy volunteers (Table 2 and Figures 4 and 5). Patients with HCM demonstrated significantly less normalized MD at the septal and the lateral AVJ compared to healthy volunteers. Both the septal and the lateral AVJ of patients with HCM recoiled at significantly slower normalized maximum velocities (s^-1^) in early diastole relative to healthy volunteers. Conversely, during diastasis, AVJ motion showed significantly faster normalized velocities in patients with HCM at both the septal and lateral AVJ. We found a 16-fold difference in the VDS/MVED ratio at the lateral AVJ and a 4-fold difference at the septal AVJ. Despite the far fewer number of hypertrophied segments and no evidence of LGE in the lateral wall, there were no statistically significant differences between the septal and lateral AVJ motion variables within the HCM group. Similarly, when comparing septal to the lateral AVJ motion variables within the healthy volunteers, we observed no significant differences. Twenty of 24 (83%) HCM exhibited LGE. Comparing HCM patients with and without LGE, we found no statistically significant differences in any of the AVJ motion variables (MD, MVED, VDS and VDS/MVED) at either septal or lateral AVJ. In comparison to healthy volunteers however, both HCM patients with and the four patients (17%) without LGE demonstrated statistically significant differences in MVED, VDS and VDS/MVED (Table 3 and Figure 6) at both the septal and lateral AVJ. Our results demonstrate good reproducibility, based on normal volunteer data intraclass correlation coefficients obtained by two independent observers of 0.84 (p < 0.001), 0.92 (p < 0.001), 0.84 (p = 0.001) and 0.79 (p = 0.006) for MD, MVED, VDS and VDS/MVED, respectively. Intra-study reliability analysis revealed intraclass correlation coefficients of 0.84 (p < 0.001), 0.55 (p = 0.003), 0.78 (p < 0.001) and 0.47 (p = 0.023) for MD, MVED, VDS and VDS/MVED, respectively.

**Table 2:** Comparison of median AVJ motion variables between healthy volunteers and HCM patients for the lateral and septal AVJ

	**Median**	**25**^**th **^**percentile**	**75**^**th **^**percentile**	**Median**	**25**^**th **^**percentile**	**75**^**th **^**percentile**	**P**
Lateral AVJ^*^	**Control**			**HCM**^**‡**^			
**MD**^**§**^	-0.149	-0.165	-0.125	-0.107	-0.121	-0.083	<0.001
**MVED**^**|| **^**(s**^**-1**^**)**	1.082	0.854	1.343	0.506	0.395	0.634	<0.001
**VDS**^**¶ **^**(s**^**-1**^**)**	0.012	-0.008	0.037	0.067	0.047	0.078	<0.001
**VDS/MVED**	0.009	-0.006	0.037	0.141	0.073	0.166	<0.001
**Septal AVJ**	**Control**			**HCM**			
**MD**	-0.149	-0.173	-0.130	-0.116	-0.130	-0.100	<0.001
**MVED (s**^**-1**^**)**	0.947	0.628	1.033	0.537	0.406	0.635	0.001
**VDS (s**^**-1**^**)**	0.027	0.004	0.060	0.074	0.040	0.109	0.006
**VDS/MVED**	0.029	0.004	0.056	0.126	0.090	0.249	<0.001

^*^AVJ = atrioventricular junction; ^‡^HCM = hypertrophic cardiomyopathy; ^§^MD = maximum displacement; ^||^MVED = maximum velocity early diastole; ^¶^VDS = velocity diastasis.

**Table 3:** Comparison of median AVJ motion variables between healthy volunteers and HCM patients with and without LGE

**Lateral**	**P**	**P**
AVJ^*^	**HCM**^**‡ **^**LGE**^**§**^**+**	**HCM LGE-**
	**vs**	**vs**
	**Healthy Volunteers**	**Healthy Volunteers**
**MD**^**||**^	<0.001	NS
**MVED**^**¶ **^**(s**^**-1**^**)**	<0.001	0.001
**VDS**^**# **^**(s**^**-1**^**)**	<0.001	0.003
**VDS/MVED**	<0.001	0.001
**Septal**	**P**	**P**
**AVJ**	**HCM LGE+**	**HCM LGE-**
	**vs**	**vs**
	**Healthy Volunteers**	**Healthy Volunteers**
**MD**	<0.001	NS
**MVED (s**^**-1**^**)**	0.001	0.032
**VDS (s**^**-1**^**)**	0.005	0.045
**VDS/MVED**	<0.001	0.023

^*^AVJ = atrioventricular junction; ^‡^HCM = hypertrophic cardiomyopathy; ^§^LGE = late gadolinium enhancement; ^||^MD = maximum displacement; ^¶^MVED = maximum velocity early diastole; ^**#**^VDS = velocity diastasis.

## Discussion

### AVJ motion tracking

We have demonstrated the feasibility of a novel and practical CMR technique, developed in our laboratory, to track longitudinal AVJ motion throughout the cardiac cycle. Using this technique, several AVJ motion variables relating to LV function can be measured. While novel to CMR, these variables are derived from principles relevant to assessment of systolic and diastolic function by established echocardiographic measurements.

The MD, measured from the displacement-versus-time curve at end systole, reflects longitudinal mitral annular excursion and corresponds to echocardiographic MAPSE.

Since mitral annular excursion occurs with longitudinal LV contraction and MAPSE predicts EF [11], the MD variable provides a measure of regional, longitudinal, as well as global LV function. To account for variability in LV size, we normalized the MD variable by the longitudinal LV length. Since the heart apex remains approximately stationary throughout the cardiac cycle, the normalized MD corresponds approximately to the maximum systolic strain derived by tissue Doppler echocardiography. The general shape of the observed AVJ displacement-versus-time curve resembles the expected strain-versus-time and LV volume-versus-time curves.

We also measured MVED, the maximum normalized velocity of the AVJ during the early, passive phase of diastole. This variable is measured from the AVJ normalized velocity-versus-time curve, which is derived by measuring the slope between sequential time points on the normalized displacement-versus-time curve. As we previously showed, MVED represents a statistically significant CMR correlate of mitral annulus E’ measured by tissue Doppler echocardiography [5]. Since we normalized the displacement measurements to the end-diastolic longitudinal LV length, MVED also approximately corresponds to maximum early diastolic strain rate measured by tissue Doppler. Consequently, the general shape of the AVJ velocity-versus-time curve resembles the expected strain rate-versus-time curve. In contrast to strain and strain rate measured by tissue Doppler, which detects differences in velocity between two adjacent areas of myocardium, AVJ motion variables reflect changes in position of a single material point. The AVJ can be accurately tracked throughout the cardiac cycle, given the excellent spatial resolution of CMR and the clear signal intensity difference between myocardium and fat in the adjacent atrioventricular groove.

We also measured the normalized velocity of the AVJ in diastasis, VDS, by finding the best-fit line through the normalized velocity-versus-time curve during the quiescent phase of diastole. The composite index VDS/MVED was calculated to accentuate the individual differences in the AVJ motion variables we noted when comparing patients with HCM to healthy volunteers.

### AVJ motion variable comparison

While all HCM patients in our study demonstrated normal or increased EF, they showed decreased MD for both the septal and lateral AVJ. As the MD corresponds to longitudinal mitral annular excursion of a single point at the AVJ, this may reflect altered regional rather than global systolic function. Similar discrepancies between global and regional systolic function had previously been observed used tagged-CMR sequences in patients with HCM [9]. In a pediatric population with HCM, Ganame et al. [12] demonstrated regional differences in strain, although global measures of systolic function were preserved. Reductions in longitudinal shortening in HCM may be compensated for by an increase in LV torsion [13], resulting in preserved global systolic function. When comparing the septal versus lateral AVJ MD within the HCM group, we found no significant differences despite the relative sparing of hypertrophy and fibrosis in the lateral wall. Our findings therefore suggest that regional systolic dysfunction may be present in patients with HCM, independent of hypertrophy, fibrosis and global LV function.

As expected, we noted slower velocities of the septal AVJ in HCM patients compared to healthy controls. Somewhat unexpectedly, however, we also found slower velocities of the lateral AVJ, despite the relative sparing of hypertrophy and fibrosis in this region. When comparing the septal and lateral AVJ velocities within the HCM group, we again found no significant differences. These findings agree with the significantly lower septal and lateral E’ velocities noted by Severino et al. [14] when comparing individuals with HCM to healthy controls. Another echocardiographic study demonstrated that individuals with certain genotype (+) HCM, but without morphologic evidence of LVH, exhibit significantly lower systolic and early diastolic mitral annulus tissue Doppler velocities compared to healthy controls [8]. These data support a hypertrophy-independent mechanism for regional systolic and diastolic dysfunction in patients with HCM. The diffuse increase in collagen content of HCM hearts [15], even in the absence of focal fibrosis identified by LGE, may contribute to myocardial dysfunction, as we observed in the lateral wall segments of our patient cohort. However, the four HCM patients without LGE also demonstrated statistically significant differences in MVED, VDS and VDS/MVED, at both the septal and lateral AVJ, compared to healthy volunteers. These results further suggest that myocardial dysfunction may occur in the absence of fibrosis detected by LGE. The small sample size of HCM patients without LGE likely precluded the demonstration of statistically significant differences in MD. In contrast to HCM disease models implicating abnormal myocyte contractility as the primary disturbance, an alternative ‘energy compromise’ hypothesis has been proposed. Crilley et al. [16] demonstrated that abnormalities in myocyte energy metabolism can occur in three different HCM genes, independent of LVH, and therefore may affect energy-dependent processes, such as the active relaxation phase of diastole. Impaired cardiomyocyte calcium handling, resulting from alterations in a variety of signal transduction pathways, can affect diastolic function as well [17].

We noted significantly higher normalized velocities of both septal and lateral AVJ during diastasis in HCM patients, indicating the extension of relaxation and passive filling into the normally quiescent phase of diastole. Elevations in left atrial pressure may account in part for the persistence of blood flow across the mitral annulus during diastasis, and the correspondingly higher AVJ velocities in this phase of diastole. The higher velocities could also represent a prolongation of the initial, normally rapid relaxation phase of early diastole into the normally quiescent period of mid-diastole. The increased mid-diastolic velocities may be analogous to the L wave noted on mitral inflow in certain patients with elevated filling pressures. Interestingly, a small subset of patients noted to have triphasic mitral inflow by Ha et al. [18] carried the diagnosis of HCM.

### Limitations

Our retrospective proof-of-concept study has several limitations, including a small sample size of HCM patients, which included only 4 women (17%). The cardiac MR studies performed on healthy volunteers did not include short axis cine images, so LV EF, although qualitatively normal, was not quantified. Although we previously demonstrated a statistically significant correlation between MVED and tissue Doppler echocardiography E’, AVJ motion and echocardiography variables were not directly correlated in this HCM cohort. However, the known E’ velocity reduction in patients with HCM, in conjunction with the decreased MVED we found associated with HCM and the two previous studies correlating E’ and MVED, indicate the relevance of AVJ motion tracking findings in HCM.

## Conclusions

Atrioventricular junction motion tracking, a practical technique developed in our laboratory, assesses mitral annular excursion and recoil by CMR. Using this novel approach, we observed highly statistically significant differences in AVJ motion variables in HCM patients compared to healthy volunteers. Even with relative sparing of hypertrophy and LGE, the morphologically normal-appearing HCM lateral wall also showed abnormal AVJ motion. Atrioventricular junction motion analysis can be performed efficiently on cine sequences that are obtained in nearly all CMR evaluations, without the need for additional acquisition time.

## Abbreviations

AVJ: Atrioventricular junction(s); CMR: Cardiovascular magnetic resonance; EF: Ejection fraction; HCM: Hypertrophic cardiomyopathy; IQR: Interquartile range; LA: Left atrium; LGE: Late gadolinium enhancement; LV: Left ventricle/ventricular; LVH: Left ventricular hypertrophy; MAPSE: Mitral annular plane systolic excursion; MD: Maximum displacement; MVED: Maximum velocity early diastole; NS: Not significant; SSFP: Steady state free precession; VDS: Velocity diastasis.

## Competing interests

The authors declare that they have no competing interest.

## Authors’ contributions

SGS helped conceive the study design, analyzed the results and wrote the manuscript. SC and SB co-wrote the atrioventricular junction motion tracking software and applied their program to obtain all data analyzed. RD, MBS, MS and SDK helped conceive the study design, interpret the data and critically revise the manuscript. LA developed the technique of atrioventricular junction motion tracking and served as the Principal Investigator for the study. All authors read and approved the final manuscript.
